# Advancing women's health through equity in quantitative sciences: promoting sex- and gender-based modeling in clinical trials and real-world studies

**DOI:** 10.3389/fdgth.2026.1811259

**Published:** 2026-06-10

**Authors:** Grammati Sarri, Michelle Hoiseth

**Affiliations:** 1 Innovative Statistics, Evidence, Value, Access and Health Policy, Cytel, Inc, London, United Kingdom; 2 Project Based Analytic Services, Cytel, Inc, Cambridge, MA, United States

**Keywords:** analytics, biostatistics, gender health disparities, sex, women health

## Introduction

Women's health issues have long been overlooked due to biased data, chronic underinvestment in female-focused research, and insufficient policy to quantify and address the gender health gap (1). Science, medicine, and health policy – all historically male-centric in purpose and execution – have been shaped by patriarchal norms and male-dominated leadership. Biomedical research has preferentially been conducted by men, on men, and for men, with women's bodies relegated to secondary status as deviations or complications rather than equally essential reference points. As recently as 2021, a collection of studies determined that patriarchal systems of power still shaped the health and well-being of most women worldwide, underscoring the urgency to upend this hierarchy (2). Despite their significant prevalence, for example, conditions such as endometriosis, polycystic ovary syndrome, chronic pain disorders, autoimmune diseases, and menopause-related health needs remain underdiagnosed, underfunded, and poorly understood (3). The ability to successfully address these disparities relies heavily on intersectoral actions that promote a coordinated effort across society and beyond the healthcare sector; this includes promoting women in leadership healthcare decision-making roles and supporting women's education and economic independence to be able to advocate for their right to equitable healthcare (1).

Women's health is not confined to conditions driven by XX chromosome biology (i.e., gynecological and pregnancy-based indications). It is an overarching term that includes conditions that disproportionally affect women (e.g., autoimmune diseases) compared with men (higher prevalence or incidence), or present differently in women than men (e.g., coronary artery disease) (4). Thus, the influence of sex (biological attributes such as hormones, chromosomes, and reproductive anatomy) and gender (social roles/norms, behaviours, and identifies) are integral to women's health, as these concepts interact with factors like genetics, race, socioeconomic status, disability, and age to shape health and disease outcomes.

## Current challenges in women's health research

Gaps in our understanding of the impact of sex and gender in health outcomes have profound consequences for women (5). Too often, women's health issues are misdiagnosed (e.g., in cardiovascular disease, attention deficit/hyperactivity disorder, and multiple sclerosis) or missed altogether. In addition, the lack of sex-specific treatment guidelines means that women are receiving unnecessarily high doses leading to more adverse effects, or inappropriate treatment, as seen in the under prescription of pain medications for women (6, 7).

However, the move away from the longstanding and predominant focus on the male body as the foundation to generate evidence, standards of care, and treatment pathways is still in its infancy. Recent advancements in trial design, analytical methods, and regulatory frameworks can facilitate the generation of sex-sensitive evidence to support regulatory and payer decision-making.

The rigorous integration of sex as a biological variable is a fundamental requirement for modern biomedical research, as medical products and interventions often stimulate distinct responses in women compared with men due to intrinsic factors like genetics, hormones, and physiology (8). Historically, women were under-represented in clinical studies, leading to a significant lack of available data regarding the safety and effectiveness of many medical treatments for female patients (9). Regulatory reinforcement on the inclusion of women in clinical research is only 28 years old (The Food and Drug Administration [FDA] Demographic Rule (21 CFR 314.50 and 601.2) (10); the National Institutes of Health policy on treating sex as a biological variable in preclinical and clinical research is only a decade old (11). Collecting sex-disaggregated data is essential for generating accurate, inclusive, and equitable health evidence. As Schubert et al. said “*To Address Women's Health Inequity, It Must First Be Measured*” (12). The opportunity to close the women's health gap starts with ensuring sex diversity in clinical trials to create sex- and gender-sensitive evidence. While regulatory bodies have increased policies on women in clinical research over the last 5 years, including the FDA's draft guidance on the Study of Sex Differences in 2025 (13), the proportions of women represented in trials are widely misaligned with the prevalence or incidence for a given health condition; however, equal trial representation does not amount to a mere 50/50 split of males and females (14). Indeed, it has been suggested that today's data collection prioritizes speed, simplicity, and average effects over equity and real-world evidence (15). Developing international, consistent definitions of sex and gender data — and establishing their routine, standardized collection through interconnected data infrastructures — are essential steps toward systematically addressing health disparities for women and girls for lifespan measurement (16, 17). Valid inferences about sex-based differences rely on appropriate selection of trial participants, biostatistical (quantitative) methods, and innovative study design, as well as appropriate data collection and processing (18). However, attempts to equalize inequity must not simultaneously introduce bias (18). Conditions that predominate in one sex or another (e.g., breast cancer in women) should not be subject to artificially balanced datasets, lest the relative prevalence of the condition be overestimated in the lower prevalence group, leading to misleading models of risk distribution.

The requirement to treat sex as a biological variable introduces several additional data management and analytical considerations beyond the basics of study design (19). The challenges in data collection stem not only from issues of representation but from a broader failure of quantitative methods' applications. Innovative data modelling and trial design that challenges the traditional parallel, double blind, placebo controlled should be prioritized (15). Modelling and simulation approaches could help optimize study planning and increase the knowledge gained (20). Leveraging artificial intelligence (AI) could be a crucial partner to those designing with innovation; however, we must be mindful of the potential consequences of AI integration, such as algorithm bias, where automated systems may unintentionally prioritize cost efficiency over patient-centered care (21). Further, as AI models are trained on historical data – which can include biases or gaps, such as underrepresentation of women and specific racial groups – an over-reliance on these models can perpetuate existing inequities (21, 22).

**Figure 1:**
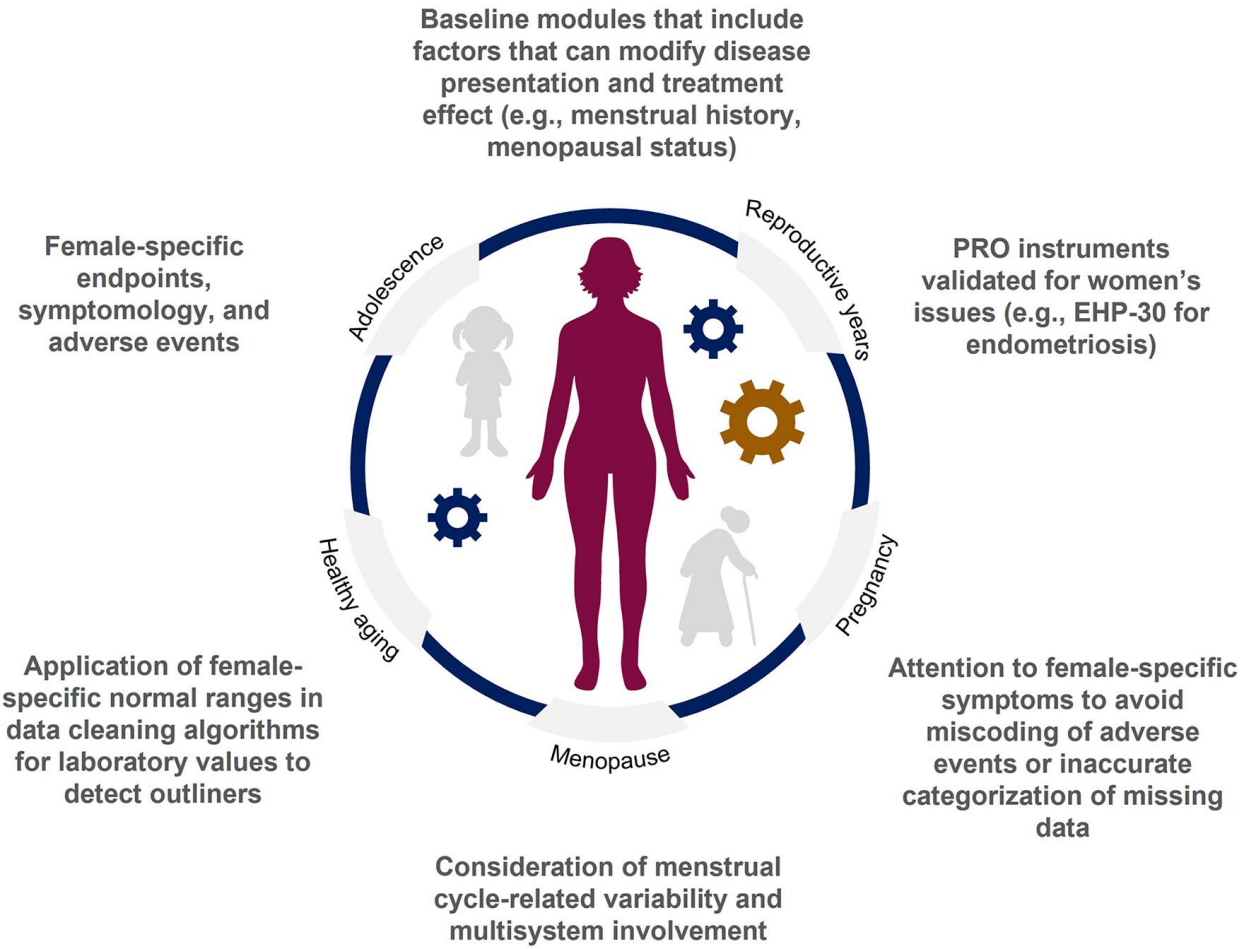
Sex- and gender-based considerations in biostatistics and clinical data management.

In the following section, the authors present specific practices for sex- and gender-informed modeling for clinical and real-world evidence generation along recommend actions (short, middle, and long-term) that can be made in the context of research and clinical practice versus what requires a system-wide commitment (Table 1, Figure 1). We encourage researchers to adhere to Sex and Gender in Research (SAGER) guidelines (23) and tools (24) to improve the quality of data in clinical research and refer to previous publications exploring technical information in these practices (13–15, 25).

**Table 1:** Recommended actions to promote sex- and gender-based modeling in clinical trials and real-world studies.

Timeline	Recommended Action	Key Concept/Requirement
Short-term (implementation & compliance)	Factor sex as a biological variable into the design, analysis, and reporting of all clinical studies.	Researchers must provide strong justification for any study proposing to use only one sex.
	Expand inclusion criteria to include women of childbearing potential in phase 1 and early phase 2 trials, when appropriate.	Focus on safe trial conduct through pregnancy monitoring and contraception counseling rather than exclusion.
	Adopt group-specific imputation for missing data; perform multiple imputation separately by randomized group (and potentially sex).	This ensures that interaction terms involving sex are not biased toward the null during the analysis.
Middle-term (advanced analytical modeling)	Integrate biological factors into pharmacokinetics modeling, such as the stages of the menstrual cycle and exogenous hormonal therapy.	Sponsors should also consider the drug's effect on the pharmacokinetics of oral contraceptives.
	Perform robust sensitivity analyses using MNAR (missing not at random) modeling to test the validity of treatment effects across sexes.	Extensive sensitivity testing should include pattern mixture, selection, and shared parameter models to account for non-random dropout.
	Analyze multivariable interactions between sex and other sociodemographic variables like age and race to detect clinically significant differences.	Moving beyond binary sex analysis to understand how intersectional variables influence drug response.
	Investigate novel dosing strategies and gather sex- and gender-sensitive data throughout the product lifecycle	
Long-term (policy & health equity)	Advance precision public health by tailoring community-level interventions based on unique biological and social profiles.	This shifts the focus from population averages to maximizing health benefits for specific community subgroups.
	Transition to precision medicine informed by unique genetics and sex-based metabolic responses to prevent drug toxicity.	Modeling should predict whether a treatment will be effective or produce specific adverse reactions based on individual biological markers.
	Establish transdisciplinary frameworks that partner with communities to address environmental health disparities affecting women.	Long-term research should utilize large sets of population data to quantify how cumulative environmental stressors impact health equity.

## Sex- and gender-informed modeling for clinical and real-world evidence generation

### Statistical power and trial design

- **Lock the Clinical Claim and Estimands:** Depending on the claim ─ all comers versus sex specific – target the population average treatment effect while modeling sex explicitly or power women for confirmatory inference, respectively. For dual claims (men and women), intersection-union or gatekeeping/alpha-split approach may plan for the maximum of per-sex sample sizes.
- **Epidemiology-based Powering:** Sample size calculations for women should be based on actual **epidemiological prevalence** or incidence rather than an arbitrary 50/50 split, acknowledging that historical biases may have obscured true disease presentation.
- **Outcomes selection:** When disease manifestation or symptom expression differs by sex and gender, several established analytic and design strategies may be used to support comparability across sexes without obscuring clinically important sex-related differences, including:
  - use of a common clinical endpoint with sex included as a design and analysis factor (e.g., stratification or interaction testing);
  - composite or responder endpoints incorporating sex-and gender-appropriate components that reflect clinically meaningful benefit in each group;
  - application of sex-specific cut points, such as differing minimally important differences (MIDs) to define response; and
  - use of latent variable or severity score models that integrate multiple correlated outcomes into a single construct while allowing measurement properties to vary by sex.
- **Analysis models:** different analytical strategies should be tailored to the type of outcomes (continuous, binary/responder, time to event). For interaction analyses, researchers should utilize full interaction models to estimate the effect of sex and gender on treatment response. Relying solely on separate subgroup *p*-values is insufficient, as a lack of statistical significance does not always equate to a lack of a clinically meaningful difference.
- **Meta-Analysis of Individual Patient Data (IPD):** Pooling IPD across multiple trials allows researchers to overcome sample size limitations in specific sex-based subgroups and explore how sex and gender may interact with intersectional factors like ethnicity, age, and disability and other social factors. Within-group differences between sexes/genders must be considered.
- **Statistical Efficiency in Missingness:** While multiple imputation is popular, for univariate outcomes, **complete case analysis (CCA) with covariate adjustment** can sometimes be more efficient and provide unbiased estimates if the analysis model is correctly specified.

### Pharmacometric and biological modeling

- **Physiologically-Based Pharmacokinetic (PBPK) Modeling:** These mechanistic frameworks simulate female-specific physiological complexities, such as slower gastric emptying (impacting drug absorption) and a higher percentage of body fat (impacting the distribution of lipophilic drugs).
- **Accounting for Hormonal Fluctuations and Longitudinal Modeling:** Models must account for time-varying exposures across a woman's lifespan, including the menstrual cycle, pregnancy, and menopause. For instance, pregnancy drastically increases plasma volume and glomerular filtration, which can reduce the concentration of renally cleared drugs.
- **Enzymatic Activity:** Quantitative models must reflect sex-based divergence in metabolic clearance, such as higher CYP3A4 activity in females compared to higher CYP1A2 in males.

### Quantifying socio-cultural gender factors

- **Composite Gender Scores:** To capture gender as a multidimensional construct, researchers are moving toward **gender scores** or composite indices. These are typically derived from variables like domestic roles (hours of housework), economic participation (breadwinner status), and psychosocial factors (risk-taking behaviour).
- **Dimension Reduction Techniques:** Statistical methods like **Principal Component Analysis (PCA)** and logistic regression are used to identify relevant variables and minimize redundancy across highly correlated gender measures into a single interpretable score.

### Advanced modelling for real-world evidence (RWE)

- **Target Trial Emulation (TTE):** This systematic approach uses observational real-world data (such as electronic health records) to mimic the protocol of a hypothetical randomised controlled trial (RCT). TTE can estimate causal effects in broader, more diverse female populations that are often excluded from traditional RCTs.
- **Digital Twins:** These multiscale computational representations of individuals use machine learning and historical health data to serve as models for female-specific physiology (e.g., the female pelvic floor or brain changes during menstruation). By simulating trial enrollment and eligibility criteria, they provide quantitative metrics on expected representation and can identify subgroups that may be inadvertently excluded under existing study design, allowing for iterative adjustment of eligibility criteria (26)
- **Structural Causal Models (SCM):** Using Directed Acyclic Graphs (DAGs) allows researchers to separate the Total Causal Effect (TCE) of sex or gender from other confounding variables or proxies.

### Algorithmic equity and data management

- **AI Bias Mitigation:** In the era of healthcare AI, quantitative researchers should apply preprocessing mitigation (such as resampling data to balance positive samples across groups), in-processing (fairness constraints in ML algorithms), and post-processing (adjusting predictions to ensure equal opportunity metrics).
- **Standardised Data Collection:** Routine data collection must be formalised for sex-disaggregated data, including specific variables on exogenous hormones (e.g., contraceptives, hormonal replacement therapy) and reproductive cycle stages.
- **Simulation Analyses:** These can be used to explore how unmeasured variables (missing biological or social factors) might influence or distort observed associations between sex/gender and health outcomes.

## Discussion

Despite growing evidence demonstrating the significant influence of sex and gender on health outcomes and encouraging country-specific regulatory progress in promoting studies' sex diversity, major gaps persist in the systematic integration of sex and gender considerations into pharmaceutical research, development, and regulatory frameworks. Resolving the women's health gap is more than just a numbers game. Quantitative integration of women's health into clinical trials and real-world studies requires moving beyond a male default paradigm to treating sex and gender as foundational scientific variables. This entails prospectively designing studies with sex balanced recruitment, stratified randomization, and power calculations that support sex specific inference, alongside prespecified analyses that test effect heterogeneity rather than rely on pooled assumptions. Endpoints and safety evaluations must reflect outcomes relevant to women across the life course, while real world analytic frameworks should address sex related differences in access, care pathways, and data completeness using bias detection and causal methods. Embedding these principles into statistical plans, reporting standards, and governance structures ensures that evidence generation is both scientifically valid and equitable. Ultimately, redesigning biostatistics with a diversity lens is essential to produce clinical evidence that accurately represents—and meaningfully benefits—the entire population.
